# Chitosan-Supported ZnO Nanoparticles: Their Green Synthesis, Characterization, and Application for the Removal of Pyridoxine HCl (Vitamin B6) from Aqueous Media

**DOI:** 10.3390/molecules29040828

**Published:** 2024-02-12

**Authors:** Samah Ali, Marwa Dayo, Sana Alahmadi, Amr Mohamed

**Affiliations:** 1 Chemistry Department, College of Science, Taibah University, Al-Madinah Al-Munawarah 42353, Saudi Arabiasahamade@taibahu.edu.sa (S.A.); 2 The National Organization for Drug Control and Research, Giza 12622, Egypt; 3 The Higher Institute of Optics Technology (HIOT), Heliopolis, Cairo 17361, Egypt

**Keywords:** pyridoxine HCl, ZnO NPs, chitosan, chicory, composite

## Abstract

A composite of chitosan-supported ZnO nanoparticles (ZnO/CS) was green-synthesized via an easy and cost-effective method using Chicory (*Cichorium intybus*) plant extract. The synthesis was confirmed using uv-vis spectrometry at a λ_max_ of 380 nm, and the surface of the material was characterized via FT−IR spectroscopy, and finally via SEM, which confirmed the distribution of ZnO nanoparticles on the surface of chitosan biopolymer (CS). The synthesized material was applied in the adsorptive removal of residues of the pyridoxine hydrochloride (vitamin B6) pharmaceutical drug from aqueous media using the batch technique. The material’s removal capacity was studied through several adjustable parameters including pH, contact time, the dose of the adsorbent, and the capacity for drug adsorption under the optimal conditions. Langmuir and Freundlich isotherms were applied to describe the adsorption process. The removal was found to obey the Freundlich model, which refers to a chemisorption process. Different kinetic models were also studied for the removal process and showed that the pseudo-second-order model was more fitted, which indicates that the removal was a chemisorption process. Thermodynamic studies were also carried out. The maximum removal of vitamin B6 by the nano-ZnO/CS composite was found to be 75% at optimal conditions. The results were compared to other reported adsorbents. Reusability tests showed that the nano-ZnO/CS composite can be efficiently reused up to seven times for the removal of PDX drugs from aqueous media.

## 1. Introduction

Materials that have extremely small particles and at least one exterior dimension in the nanoscale (between 1 and 100 nm) are known as nanomaterials (NMs). NMs are interesting due to their extraordinary and distinctive characteristics, including their high surface reactivity and surface-area-to-volume ratio, enhanced support for catalysts, and exceptional mechanical, optical, and magnetic properties [1], as well as their strong adsorption abilities that enable their use in the adsorptive removal of pharmaceuticals, heavy metals, and dyes from aqueous media, including wastewater [2]. Additionally, nanomaterials have the capability to enhance or establish new properties in polymers when combined with them, including optical, thermal, magnetic, and electrical characteristics [3]. Nanomaterials dispersed in a polymer matrix are called polymer composites, and these have recently gained great research interest due to their potential applications in several scientific and technological domains [4].

Chitosan (CS) is a natural polysaccharide polymer, a deacetylated product of chitin, which is the second most prevalent natural polymer after cellulose. It can be obtained from natural bio-sources like shrimp and crab shells [5]. CS possesses unique properties like its biocompatibility, high biodegradability, low toxicity, natural availability, and low cost, which make it suitable for many applications, and most importantly, for its use in wastewater treatment [6]. Because of the presence of amine and hydroxyl groups on the backbone of chitosan, it can be easily adapted to improve its characteristics or add new ones. Chitosan can be modified via chemical methods, like grafting and crosslinking, or physical methods like blending with other materials. The modification of chitosan with NMs, specifically metal oxide nanoparticles such as the oxides of zinc, copper, titanium, and iron, has recently gained considerable attention due to the exceptional value added to CS [7,8].

Zinc oxide nanoparticles (ZnO NPs) are amongst the most widely used nanomaterials in various fields due to their distinct properties such as their biocompatibility, abundant availability, photoactivity, and low production costs [9]. Recently, nano-ZnO/CS composite has been reported as a material with many applications such as a biocatalyst [10], in food packaging [11], in textiles [12], in dye removal [13], and in the removal of heavy metals [14].

Pharmaceuticals are either natural or synthetic substances which are considered hazardous environmental contaminants due to the active and complex compounds present in their formulas [15]. Several types of pharmaceutical substances have been found in significant concentrations in surface, ground, and subsurface water, domestic wastewater, municipal wastewater, and industrial effluents [16]. They are being introduced into the environment through pharmaceutical production and preparation, improper disposal, and patient use [17]. Several methods have been used for the successful removal of pharmaceuticals from aqueous media, even though this can be very challenging. Those methods include advanced oxidation processes like ozonation and photo-Fenton reactions, and adsorption using different adsorbents like activated carbon, aerogels, and magnetic nanoparticles [18].

**Figure 1:**
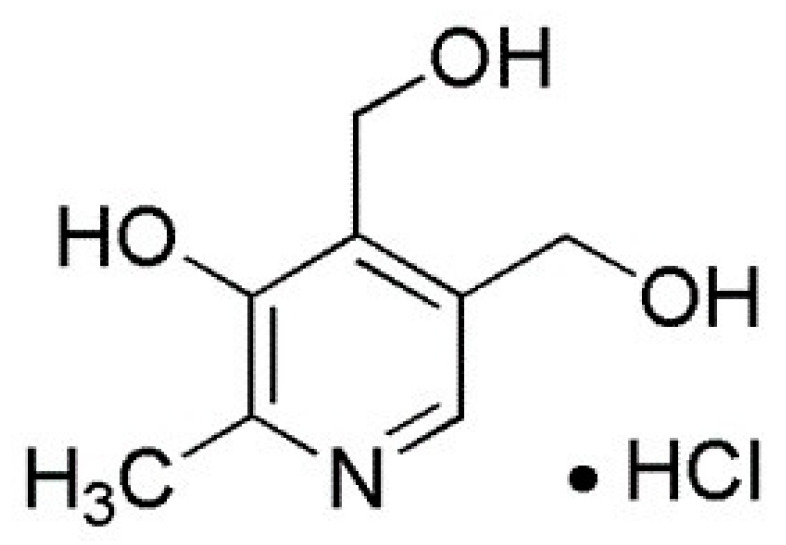
Chemical structure of pyridoxine HCl (vitamin B6) [22].

Vitamin B6 is a natural water-soluble vitamin which is vital for many living organisms as it is important for cellular processes, such as the metabolism of carbohydrates, proteins, and lipids; the biosynthesis of chlorophyll, ethylene, heme niacin, and auxin; and the regulation of transcription. Living organisms may obtain it either from biosynthesis or from food and nutrients. Vitamin B6 can also be synthetic and its main commercial form is pyridoxine hydrochloride (PDX), whose chemical structure is shown in Figure 1. PDX is the most active form of vitamin B6 and is commonly present in off-the-shelf vitamin supplements [19]. An increased dose of vitamin B6 in humans and animals leads to toxicity, which causes neuropathy [20], and its increased presence in wastewater leads to an increase in the chemical oxygen demand (COD). The higher the COD level, the greater the amount of oxidizable organic material and the lower the dissolved oxygen in water, which can cause anaerobic conditions that endanger living organisms [21]. Therefore, it is important to treat aqueous media and try to remove vitamin B6 residues using effective methods.

In this study, a nano-ZnO/CS composite was synthesized via a simple and cost-effective green method and was then applied to the removal of the pyridoxine HCl (vitamin B6) drug from aqueous media, which is the first reported application of this composite for the removal of pharmaceutical residues. Adsorption isotherm, thermodynamic, and kinetics studies were carried out for the removal process.

## 2. Results and Discussion

### 2.1. Characterization

#### 2.1.1. UV-Vis Spectroscopy

**Figure 2:**
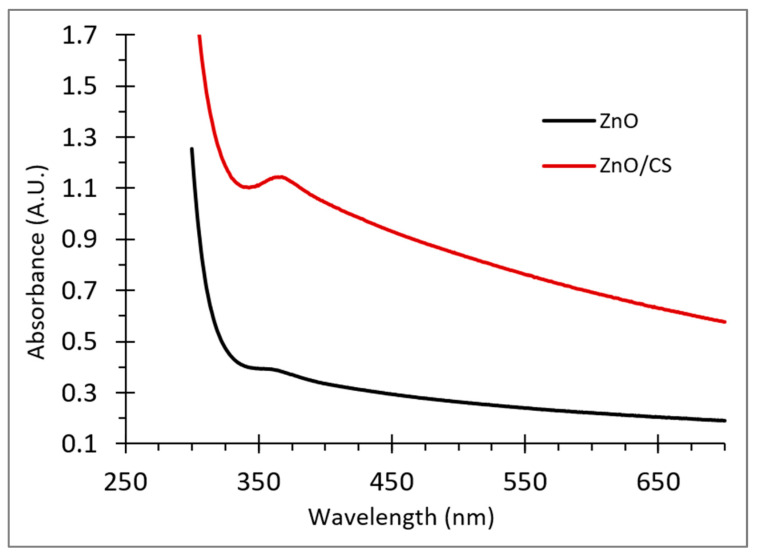
UV-vis spectra of ZnO NPs and ZnO/CS composite.

The ZnO NPs and ZnO/CS composite were initially characterized using a uv-vis spectrometer in the range of 200–800 nm. As shown in Figure 2, a broad band at 350 nm was detected for the ZnO NPs; this large absorption band is generated due to the electron jump from the valence band to the conduction band, which is mainly because ZnO is a direct-band-gap semiconductor with a wide band-gap [23]. According to previous reports, ZnO NPs possess a distinct absorption band in the range of 280–400 nm [24]. The peak of ZnO/CS was red-shifted to 380 nm, as shown in Figure 2; it has been previously reported that ZnO/CS composites exhibit a strong absorption band in the range of 350–380 nm [25]. Thus, the spectra confirm the distribution of the ZnO NPs on the chitosan surface developing the ZnO/CS composite.

#### 2.1.2. Scanning Electron Microscopy (SEM)

**Figure 3:**
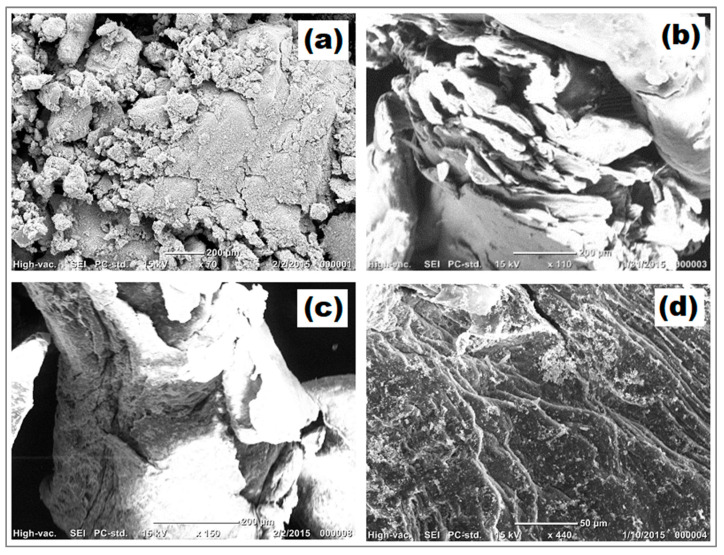
SEM images of (**a**) ZnO NPs, (**b**) chitosan, (**c**) ZnO/CS composite before adsorption, and (**d**) ZnO/CS composite after adsorption.

The surface morphology of the synthesized ZnO NPs and ZnO/CS composite were studied using scanning electron microscopy (SEM). The ZnO NPs appeared to be spherical in morphology with good distribution, as shown in Figure 3a. The ZnO/CS composite is compared with pure chitosan in Figure 3b. Chitosan showed a rough, irregular, and highly porous surface. The ZnO/CS composite in Figure 3c also shows an irregular and rough surface including micro-pores and small fractures, but the surface is filled with distributed ZnO NPs [10]. The pores that could be observed in ZnO/CS before adsorption were hardly found in ZnO/CS after adsorption, confirming the adsorption of PDX as shown in Figure 3d.

#### 2.1.3. Fourier Transform Infrared Spectroscopy (FT−IR)

**Figure 4:**
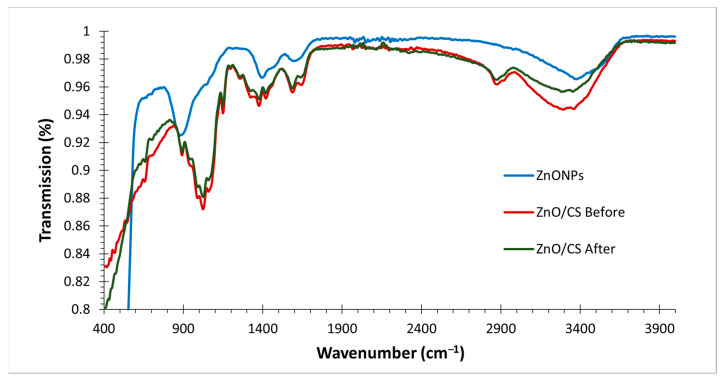
FT−IR spectra of ZnO NPs, ZnO/CS composite before, and ZnO/CS composite after adsorption.

The ZnO nanoparticles and ZnO/CS composite were characterized using FT−IR spectroscopy in the range of 400–4000 cm^−1^; the results are shown in Figure 4. The FT−IR spectrum of the synthesized ZnO NPs showed transmission bands around 653, 879, 1048, 1394, 1512, 1676, 3423, and 3553 cm^−1^. The stretching vibration of (Zn–O) was observed at 653 cm^−1^, confirming the synthesis of ZnO NPs as suggested by Shabaani et al. [26]. The bands in the range of 800–1700 cm^−1^ indicate the presence of organic compounds. Carbonyl (C=O) and (C=C) functional groups are observed at 1676 and 1512 cm^−1^, respectively. The band at 1048 cm^−1^ corresponds to (C–O). The broad band around 3423 and 3553 cm^−1^ is assigned to the stretching vibration of the amine group (N–H) and the hydroxyl group (–OH), respectively. The spectrum of the ZnO/CS composite shows bands around 657, 892, 990, 1024, 1058, 1150, 1287, 1376, 1420, 1587, 1644, 2878, 2917, 3292, and 3361 cm^−1^. The band at 657 cm^−1^ confirms the presence of ZnO NPs. The bands at 1024 and 1058 cm^−1^ correspond to the stretching vibrations of (C–O), and the peak at 1287 cm^−1^ corresponds to the stretching vibration of (C–N). The bending bands of (C–H) were observed at 1376 and 1420 cm^−1^, and the bending band of (–NH) primary amide was found at 1587 cm^−1^. The carbonyl group (C=O) observed at 1644 cm^−1^ confirms the presence of residual N–acetyl groups. The bands at 2878 and 2917 cm^−1^ are attributed to (C–H) asymmetric and symmetric stretching vibrations, respectively. The broad band around 3292 and 3361 cm^−1^ corresponds to (–NH) and (–OH) stretching vibrations. The band at 1150 cm^−1^ resembles the β (1–4) glycosidic bond in polysaccharides, and comparable results were previously reported by Queiroz et al. [27]. There was a significant difference between the ZnO/CS composite sample before and after adsorption. The spectrum of ZnO/CS in Figure 4 appears to be diminished in the intensity of bands after adsorption and slightly shifted, and a new band was observed at 2195–2350 cm^−1^, which might be due to a chemical transformation during the adsorption process [28].

### 2.2. Determination of Zero-Point Charge (PZC)

**Figure 5:**
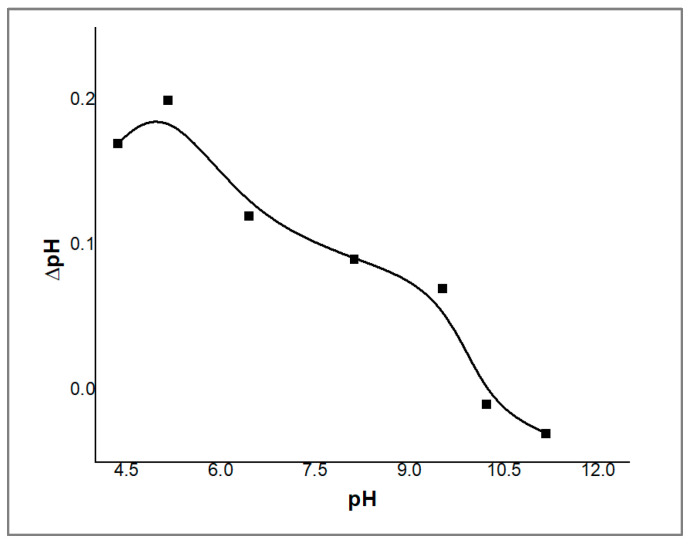
Zero-point charge of the synthesized ZnO/CS composite sorbent.

The zero-point charge (PZC) was determined to obtain information on the surface charge of the ZnO/CS composite sorbent. The ΔpH was calculated and plotted against the initial pH, and the results are shown in Figure 5. The PZC value of the ZnO/CS surface was 7, as previously reported by Yazdani et al. [29]. Thus, the sorbent is positively charged at the pH below the point-zero charge due to the adsorption of excess H^+^ and negatively charged at the pH above it due to the desorption of H^+^ [30]. The maximum removal of PDX by the ZnO/CS composite sorbent occurs at pH values above the PZC. Therefore, it is assumed that the drug cations are drawn to the negatively charged surface of the adsorbent.

### 2.3. Effect of pH

**Figure 6:**
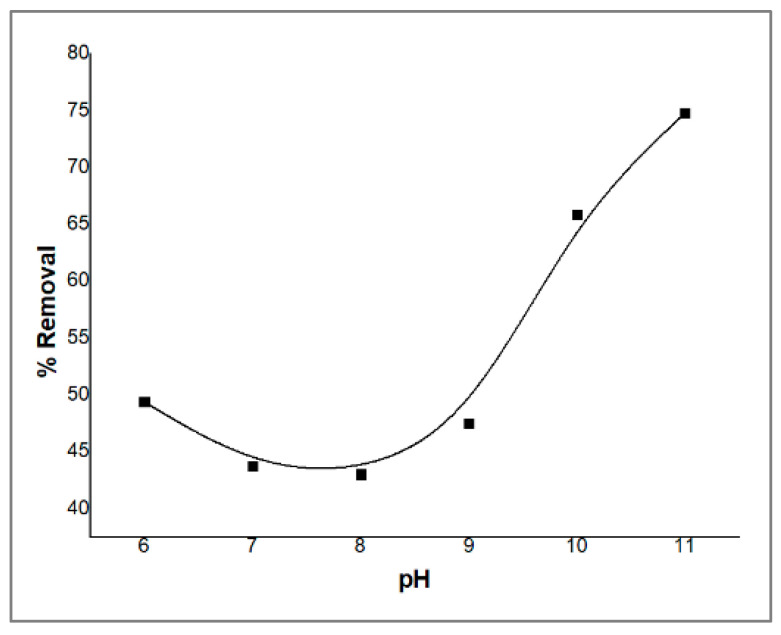
Effect of pH on the removal of PDX by ZnO/CS.

The pH is a key parameter in the adsorption process since the mechanism of the adsorption is mainly dependent on the concentration of [H_3_O^+^] or [OH^−^] ions in the solution [31]. A fixed amount of ZnO/CS composite sorbent (0.02 g) was shaken with a constant concentration of PDX solution (5 ppm) for 30 min, varying the pH by adding a buffer in the range of pH 5–11. The results of the pH effect on the adsorption of PDX are shown in Figure 6. The PDX’s removal decreased with increasing pH from 5 to 8, then increased from 8 to 11 to reach a maximal removal of 75% for PDX at a pH of 11, which shows that basic conditions are more preferable than acidic ones for elimination. This might be attributed to the p*K_a_* values of the PDX drug as well as the surface charge of the ZnO/CS sorbent. The p*K_a_* values of PDX are p*K_a_*_1_ = 5.0 and p*K_a_*_2_ = 8.96 [32]. The cationic form of PDX is present at a pH lower than 5, which decreases the removal percentage as a result of a possible electrostatic repulsion with the positive charges on the ZnO/CS sorbent. The adsorption capacities of PDX increased when the pH value increased, most probably because of the decrease in PDX’s positive species in the aqueous solution. Therefore, all further experiments were performed at a pH of 11.

### 2.4. Effect of Contact Time

**Figure 7:**
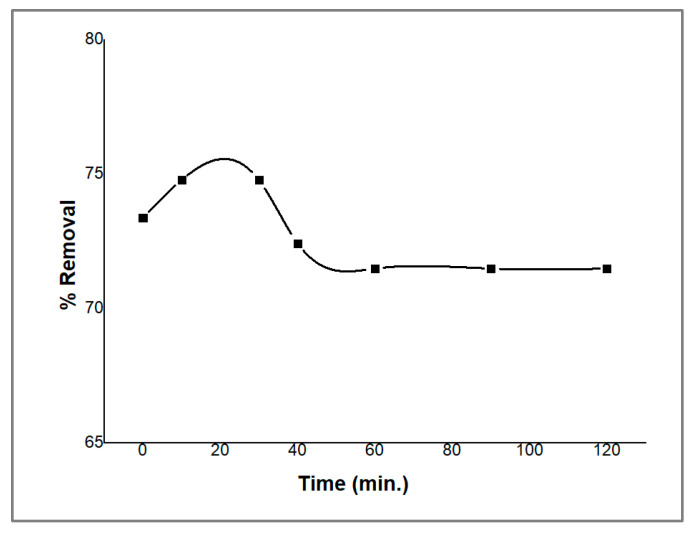
Effect of contact time on the removal of PDX by ZnO/CS composite.

The adsorption of PDX on the ZnO/CS composite was examined as a function of contact time in order to determine the equilibration time for maximal removal. The results are shown in Figure 7. The percentage removal of the PDX drug reached its maximal peak (75%) after 10 min, and it stayed the same until 30 min. After that, the removal percentage decreased to reach equilibrium within 60–120 min. The decrease in adsorption is probably due to the rapid occupation of active sites on the ZnO/CS composite surface in the beginning, leading to the decrease in unoccupied active sites which causes repulsive forces between the absorbed ions of the PDX drug and the remaining ions in the solution, delaying the equilibrium time [33]. These results indicate that a period of 30 min is the optimal contact time for the removal of the PDX drug from aqueous media.

### 2.5. Effect of Sorbent Amount

**Figure 8:**
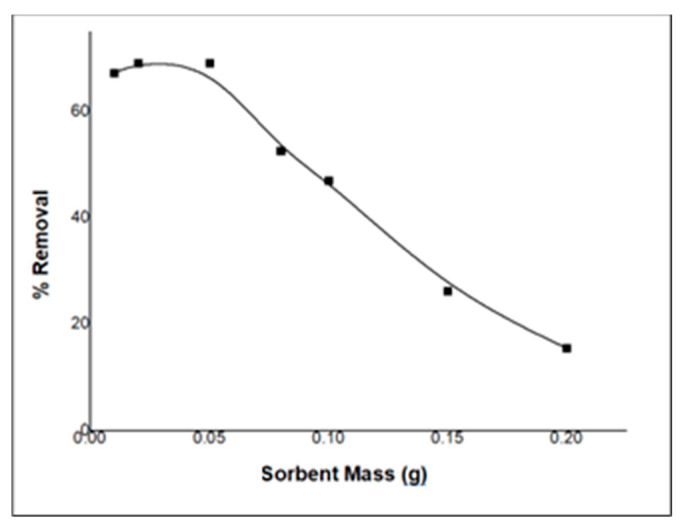
Effect of the amount of sorbent on the removal of PDX drug by ZnO/CS.

To examine the effect of the amount of sorbent on the adsorption process, experiments were carried out by varying the sorbent amount from 0.01 to 0.2 g. As shown in Figure 8, the initial removal of PDX of 5 ppm increased slightly to reach its maximum at 0.02 g, after which it started to decrease with the increasing amount of sorbent. This could be due to the agglomeration of composite particles which causes a decrease in the effective surface area of the sorbent and sequentially reduces the adsorption of PDX drug particles [13].

### 2.6. Kinetic Models

**Figure 9:**
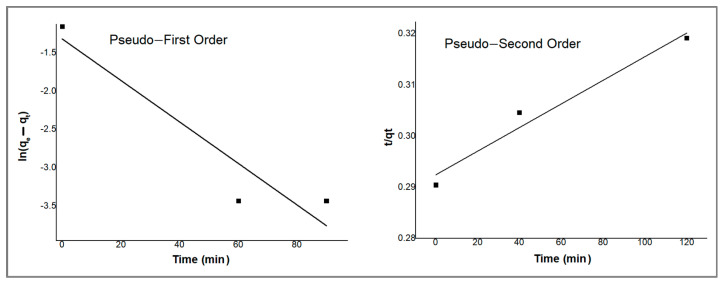
Pseudo-first-order and pseudo-second-order kinetic models for the removal of PDX by ZnO/CS.

The mechanism of PDX drug adsorption onto ZnO/CS composite can be described by studying the rate expression using pseudo-first-order and pseudo-second-order kinetic models. The pseudo-first-order kinetic model assumes that the rate of occupation of the adsorbent’s vacant sites is proportionate to the number of unoccupied sites, which, in turn, resembles a physisorption process [34]. On the other hand, the pseudo-second-order kinetic model assumes that the rate-limiting step is chemisorption and is effective over the whole range of adsorption involving the exchange of electrons between adsorbate and adsorbent particles [35]. The linear forms of the pseudo-first-order and pseudo-second-order kinetic models are shown in Equations (1) and (2) [36,37]:

(1) $$\log\left(q_{e}-q_{t}\right)=log{\;q}_{e}-\frac{k_{1}}{2.303}t\;$$

(2) $$\frac{t}{q_{t}}=\frac{1}{k_{2}{q_{e}}^{2}}+\frac{t}{q_{e}}\;$$

where *q_e_* and *q_t_* are the adsorption capacities at equilibrium and at time *t* in mg·g^−1^, *k*_1_ is the rate constant of the pseudo-first-order adsorption process (min^−1^), and *k*_2_ is the rate constant of the pseudo-second-order adsorption process [g·(mg·min)^−1^]. The linear fit of the experimental data to pseudo-first-order and pseudo-second-order kinetic models are shown in Figure 9. The kinetic parameters and the correlation coefficients (*R*^2^) are summarized in Table 1.

The correlation coefficient (*R*^2^) value for the pseudo-second-order kinetic model is observed to be higher than the pseudo-first-order one. The kinetic data suggest that the adsorption of PDX by ZnO/CS composite is well described by the pseudo-second-order model, which presumes the tendency towards the chemisorption process [38].

### 2.7. Adsorption Isotherms

The adsorbent capacity can be described by adsorption isotherms, which can be used to study the adsorption mechanism thoroughly. The experimental data were subjected to two adsorption isotherms, namely, the Langmuir and Freundlich isotherms. The Langmuir adsorption isotherm describes the adsorption as a monolayer coverage on a homogeneous adsorption surface with a fixed number of available active sites and identical energy, where there is no interaction between adjacent adsorbed molecules [39], whereas the Freundlich adsorption isotherm describes the adsorption as a multilayer coverage on a heterogeneous adsorption surface with exponential distribution of active sites and their energies [40]. The linear forms of the Langmuir and Freundlich isotherms are represented by Equations (3) and (4) [41,42]:

(3) $$\frac{C_{e}}{q_{e}}=\left(\frac{1}{q_{m}}\right)b+\frac{C_{e}}{q_{m}}\;$$

(4) $$\log q_{e}=logK_{f}+\frac{1}{n}\;logC_{e}$$

where *q_e_* and *C_e_* are the adsorbed amount of the drug at equilibrium (mg·g^−1^) and the concentration, respectively, *q_m_* is the monolayer maximum adsorption capacity of the adsorbent in solution (mg·L^−1^), *b* is the Langmuir constant related to the free energy of adsorption, *K_f_* is a constant related to the adsorption capacity (mg·g^−1^), and *1/n* is an empirical parameter related to the adsorption intensity (where *1/n* = 0 is irreversible, *1/n* > 1 is unfavorable, and 0 < *1/n* < 1 is favorable) [43]. 

**Figure 10:**
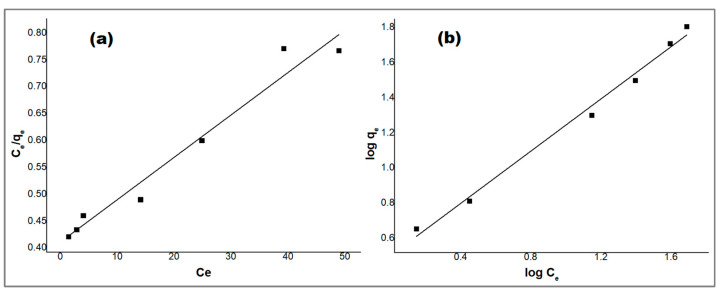
(**a**) Langmuir and (**b**) Freundlich adsorption isotherms for PDX removal by ZnO/CS composite.

The experimental data were fitted to the linear equations of the Langmuir and Freundlich isotherms as shown in Figure 10, and the isotherm parameters and correlation coefficients (*R*^2^) for the Langmuir and Freundlich isotherms are presented in Table 2.

The adsorption of the PDX drug onto ZnO/CS obeys the Freundlich isotherm model rather than the Langmuir isotherm model where the value of the correlation coefficient *R*^2^ (0.991) of the Freundlich isotherm is higher than the value of *R*^2^ (0.972) of the Langmuir isotherm, as shown in Table 2. The value of *1/n* from the Freundlich model data is 0.743, which confirms that the adsorption is favorable, and that it is a chemisorption process [44].

### 2.8. Thermodynamic Studies

**Figure 11:**
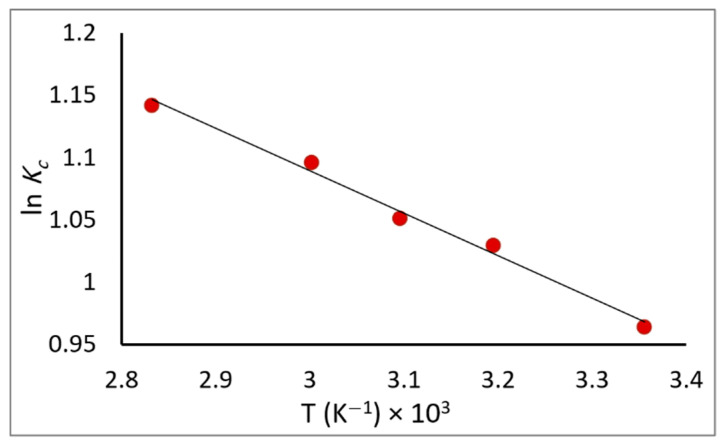
Effect of temperature on the removal of PDX by ZnO/CS.

A change in temperature can affect the adsorption process; thus, the adsorption of the PDX drug on the ZnO/CS composite was investigated within a temperature range of 298–353 K at optimal conditions. The thermodynamic parameters, including entropy ∆*S*, enthalpy ∆*H*, and Gibbs free energy ∆*G*, were determined using Equations (5) and (6) [45,46]:

(5) $$lnK_{c}=-\frac{∆H}{RT}+\frac{∆S}{R}\;$$

(6) $$∆G=-RT\;lnK_{c}\;$$

where *T*, ∆*S*, ∆*H*, and ∆*G* are the absolute temperature, entropy, enthalpy, and Gibbs free energy, respectively. *R* represents the gas constant (8.314 J·mol^−1^·K^−1^), *K_c_* = *q_e_/C_e_* is the equilibrium constant, and *q_e_* and *C_e_* are the equilibrium concentrations (mg·L^−1^) of PDX on the adsorbent and in the solution, respectively. The thermodynamic parameters are shown in Table 3, which can be calculated from the slope and intercept of the plot of *lnK_c_* versus *T* which gave a straight line as shown in Figure 11.

From the results presented in Table 3, the negative values of ∆*G* and ∆*H* suggest that the adsorption process is exothermic and spontaneous in nature. The value of ∆*G* de-creases with increasing *T*, which means that the process is more spontaneous at high temperatures [47]; this might be caused by the increase in the number of adsorption sites resulting from internal bond breaking near the adsorbent’s active surface sites [45]. Moreover, the value of entropy ∆*S* showed an increase in randomness at the adsorbent/adsorbate interface and confirms that the adsorption is favorable [48].

### 2.9. Reusability of the Sorbent

**Figure 12:**
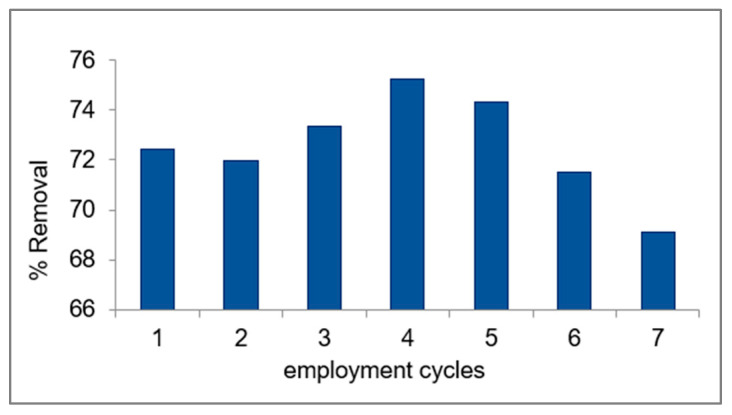
The removal percentage vs. employment cycles of nano-ZnO/CS composite for the removal of PDX drug from aqueous media.

The reusability of the nano-ZnO/CS composite for the removal of the PDX drug from aqueous media was examined by repeating the experiment under the same optimal conditions until reaching the maximum adsorption limit and a significant decrease in the percentage of removal. As shown in Figure 12, the removal percentage did not significantly decrease until the seventh time, which is most probably due to the decrease in the available active sites and pores of the sorbent after the seventh application. The maximum adsorption capacity of the nano-ZnO/CS composite was found to be 4.7 mg·g^−1^. Thus, the ZnO/CS composite works efficiently and can be reused up to seven times for the removal of the PDX drug from aqueous media [49].

### 2.10. Comparison with Other Adsorbents

A comparison was conducted between the ZnO/CS composite’s and other adsorbents’ efficiency for the removal of the PDX drug from aqueous media, and the results are shown in Table 4. It can be concluded that the ZnO/CS composite has a comparable adsorption capacity for the PDX drug, with a significantly higher removal percentage than the other sorbents at the lower PDX drug concentration (5 ppm).

## 3. Materials and Methods

### 3.1. Instrumentation

The pH of the solutions was measured by using an HI 221 pH meter (Hanna Instruments, Eibar, Spain) with Ag/AgCl electrode. A digital orbital shaker (Stuart SSL1, Carl Roth, Karlsruhe, Germany) was used for the sorption experiments, and a centrifuge (Kubota 2010, Kubota Corp., Tokyo, Japan) was used to accumulate the ZnO nanoparticles. The available spectrometer did not include an integrating sphere. The analysis was carried out using a uv-vis spectrometer (Evolution 201, Thermo Scientific, MA, USA) with matching glass cells (10 mm) by mixing the sample with KBr salt, and the measurements were recorded at a scan range of 400–4000 cm^−1^ with a resolution of 2 cm^−1^. The surface of the synthesized ZnO/CS sorbent was characterized using a scanning electron microscope (Jeol 1400, Jeol Ltd., Tokyo, Japan) and a Fourier transform infrared spectrometer (FTIR-8400S, Shimadzu, Kyoto, Japan). 

### 3.2. Materials and Reagents

All the chemical reagents used were of analytical grade. Chicory plant (*Cichorium intybus*) was obtained from a commercial store in Saudi Arabia. Chitosan was obtained from Al-Alamia Chemicals Co., Cairo, Egypt (density = 0.150 g/mL, moisture level = 8.2%). Pyridoxine hydrochloride (vitamin B6) was supplied by the Egyptian National Organization for Drug Control and Research (NODCAR), Cairo, Egypt. Double-distilled water was used to prepare all solutions. In order to create a stock solution of PDX (200 ppm), the exact amount of the drug was dissolved in double-distilled water. Drug working standard solutions were prepared using the proper dilution.

### 3.3. Preparation of Chicory (Cichorium intybus) Plant Extract

After washing with double-distilled water, the Chicory plant (*Cichorium intybus*) was cleaned of dust and other impurities, dried, and grounded. The plant extract was prepared by suspending 2.0 g of the plant powder in 100 mL of double-distilled water, and then the suspended solution was shaken at 200 rpm for 2 h. The solution was then filtered. Before usage, the filtrate was kept in a refrigerator at 4–6 °C.

### 3.4. Synthesis of Nano-ZnO/CS Composite

ZnO NPs were synthesized using the procedure previously described by Taha et al. [52], by mixing 10 mL of Chicory plant extract with 100 mL of 0.05 M zinc nitrate Zn(NO_3_)_2_ solution. The mixture was then heated and vigorously stirred using a magnetic stirrer at 70 °C for an hour with the pH adjusted to 8 by dropwise addition of 1 M NaOH solution. An off-white-to-yellow precipitate was formed at the end of the reaction. Then, a mass of 5.0 g of chitosan powder was added to the solution while shaking on a digital orbital shaker for 6 h. The suspension was left to precipitate and was then filtered. Prior to the next steps, the obtained nano-ZnO/CS composite sample was dried at room temperature after being rinsed with ethanol and distilled water. It is noteworthy that the chitosan in this procedure was not dissolved in the solution since it dissolves only in acidic media [53]. CS was intended to be a substrate for supporting ZnO NPs.

### 3.5. Batch Technique

The removal of pyridoxine hydrochloride (PDX) was performed using the batch technique at ambient temperature. A mass of 0.02 g of ZnO/CS composite was suspended in 25 mL of PDX drug (5 ppm) and shaken for 30 min at 100 rpm. After filtering the suspension, the remaining drug concentration in the mixture was determined spectrophotometrically at the λ_max_ of the PDX drug (320 nm). In order to generate the calibration curves, the absorbance of solutions of different PDX concentrations in the range of 0.02–0.25 ppm was measured against a blank. The percentage of removal was estimated using Equation (7) [54]:

(7) $$R\%=\frac{C_{0}-C_{t}}{C_{0}}\times 100$$

where *C*_0_ and *C_t_* are the initial and final concentrations of adsorbate in liquid phase (mg·L^−1^), respectively.

The dependence of the removal of PDX on the pH value was investigated in a pH range of 5–11 by suspending 0.02 g of the ZnO/CS composite in 2.5 mL solution of PDX (50 ppm) with 2.5 mL of each buffer solution and completing the volume to 25 mL, which was then shaken for 30 min at 100 rpm. The absorbance of the samples was measured against a blank at λ_max_ of PDX. The surface morphology of ZnO/CS composite was studied using a scanning electron microscope (SEM) at an accelerating voltage of 15 kV. The surface chemistry as well as the characteristic groups of ZnO/CS composite were studied using Fourier transform infrared spectroscopy (FT−IR) in the range of 500–4000 cm^−1^.

Batch experiments were conducted to study the effect of multiple parameters that might influence the removal of PDX from aqueous solution (5 ppm) by nano-ZnO/CS composite sorbent, including the pH level, the impact of extraction duration on sorption capacity over time (0–100 min), the maximum adsorption capacity of ZnO/CS composite at variable concentrations of PDX (1–100 ppm), and the effect of the amount of sorbent on the removal of PDX from water at variable amounts of ZnO/CS composite sorbent (0.01–0.2 g). 

### 3.6. Reusability of the Sorbent

For the reusability test, a mass of 0.02 g of nano-ZnO/CS composite was added to 5 mL of PDX (50 ppm) and topped up to 25 mL with deionized water. The nano-ZnO/CS composite sample was filtered after removal and washed with double-distilled water, then left to air-dry completely before reusing under the same previous conditions.

### 3.7. Determination of Zero-Point Charge of Nano-ZnO/CS Composite

The zero-point charge (PZC) of ZnO/CS was measured using the potentiometric titration technique. An amount of 0.01 g of ZnO/CS composite was suspended in 20 mL of several buffer solutions within a pH range of 4.5 to 11. The pH remained constant after 4 h of shaking at 150 rpm. Using a digital pH meter, the pH of the suspension was determined. To obtain the potentiometric curve, the equilibrium pH (ΔpH) was plotted against the starting pH values. The PZC was determined as the pH at which the lowest ΔpH value was attained.

## 4. Conclusions

In this study, a nano-ZnO/CS composite was synthesized by a simple, inexpensive, and eco-friendly method using Chicory (*Cichorium intybus*) plant extract. The synthesis was confirmed using uv-vis spectrometry, and the surface of the material was characterized by FT−IR spectroscopy. SEM images confirmed the distribution of ZnO spherical nanoparticles on the surface of chitosan biopolymer. The synthesized material was then applied as an adsorbent to remove the pyridoxine HCl drug (PDX—vitamin B6) from aqueous media. The removal percentage of PDX was 75% under the optimal conditions: pH 11, 30 min contact time, and 0.02 g of sorbent. The experimental data were described by adsorption isotherms and kinetic models. The results suggested that the adsorption of PDX follows the Freundlich model with *R*^2^ = 0.991, and that the adsorption process follows the pseudo-second-order kinetic model with *R*^2^ = 0.967, which indicates a chemisorption process. The synthesized composite adsorbent showed a significantly higher removal percentage (75%) than the previously reported sorbents at a very low PDX drug concentration (5 ppm). The reusability tests confirmed that the composite can be efficiently reused for the removal of the PDX drug from aqueous media up to seven times. From these findings, it can be suggested that the nano-ZnO/CS composite may serve as a promising eco-friendly reusable adsorbent for the efficient removal of PDX drug residues from aqueous media such as urban and industrial liquid effluents.

## Figures and Tables

**Table 1:** Kinetic model parameters of the removal of PDX by ZnO/CS.

Pseudo-First Order			Pseudo-Second Order		
*k*_1_ (min^−1^)	*q_e_* (mg·g^−1^)	*R* ^2^	*k*_2_ (g·(mg·min)^−1^)	*q_e_* (mg·g^−1^)	*R* ^2^
−2.2 × 10^−4^	0.2675	0.893	1.169 × 10^−8^	5000	0.967

**Table 2:** Parameters of Langmuir and Freundlich isotherm models for PDX drug removal by ZnO/CS composite.

Langmuir Isotherm			Freundlich Isotherm		
*q_m_*	*b*	*R* ^2^	*K_f_*	*1/n*	*R* ^2^
2.4068	0.0190	0.972	0.3156	0.7425	0.991

**Table 3:** Thermodynamic parameters of PDX removal by ZnO/CS composite.

*T* (K)	*K_c_*	∆*G* (kJ·mol^−1^)	∆*H* (kJ·mol^−1^)	∆*S* (kJ·mol^−1^·K^−1^)
298	2.622	−5.098	−2.838 × 10^−3^	1.757 × 10^−2^
313	2.799	−5.355		
323	2.862	−5.526		
333	2.993	−5.697		
353	3.133	−6.039		

**Table 4:** The efficiency of removal of PDX drug from aqueous media by nano-ZnO/CS composite sorbent vs. other reported sorbents.

Adsorbate	Adsorbent	PDX Concentration	% Removal	PDX Concentration	% Removal	Reference
Pyridoxine HCl (vitamin B6)	PUF	30.0 ppm	50.0%	5.0 ppm	20.0%	[50]
	ZnO	80.0 ppm	84.0%		10.0%	[51]
	ZnO/CS	5.0 ppm	75.0%		75.0%	This study

## Data Availability

Data available on request.
